# Potential Therapeutic Role of Dietary Supplementation with *Spirulina platensis* on the Erectile Function of Obese Rats Fed a Hypercaloric Diet

**DOI:** 10.1155/2020/3293065

**Published:** 2020-06-30

**Authors:** Anderson Fellyp Avelino Diniz, Iara Leão Luna de Souza, Elba dos Santos Ferreira, Maria Thaynan de Lima Carvalho, Bárbara Cavalcanti Barros, Paula Benvindo Ferreira, Maria da Conceição Correia Silva, Francisco Fernandes Lacerda Júnior, Lydiane de Lima Tavares Toscano, Alexandre Sérgio Silva, Fabiana de Andrade Cavalcante, Bagnólia Araújo da Silva

**Affiliations:** ^1^Postgraduate Program in Natural and Synthetic Products Bioactive/Health Sciences Center, Federal University of Paraíba, João Pessoa, Paraíba, Brazil; ^2^Department of Biological Sciences and Health, State University of Roraima, Boa Vista, Roraima, Brazil; ^3^University Center Estácio of Amazônia, Boa Vista, Roraima, Brazil; ^4^Health Sciences Center, Federal University of Paraiba, João Pessoa, Paraíba, Brazil; ^5^Postgraduate Program in Nutrition Science/Health Sciences Center, Federal University of Paraíba, João Pessoa, Paraíba, Brazil; ^6^Physical Education Department, Health Sciences Center, Federal University of Paraiba, João Pessoa, Paraíba, Brazil; ^7^Physiology and Pathology Department/Health Sciences Center/Federal University of Paraiba, João Pessoa, Paraíba, Brazil; ^8^Pharmaceutical Sciences Department/Health Sciences Center/Federal University of Paraiba, João Pessoa, Paraíba, Brazil

## Abstract

*Spirulina platensis*, an important source of bioactive compounds, is a multicellular, filamentous cyanobacterium rich in high-quality proteins, vitamins, minerals, and antioxidants. Due to its nutrient composition, the alga is considered a complete food and is recognized for its anti-inflammatory, antioxidant, antiobesity, and reproprotective effects. All of which are important for prevention and treatment of organic and metabolic disorders such as obesity and erectile dysfunction. The aim of this study was to investigate the modulatory role of *Spirulina platensis* food supplementation and the mechanisms of action involved in reversing the damage caused by a hypercaloric diet on the erectile function of rats. The animals were divided into a standard diet group (SD, *n* = 5); a hypercaloric diet group (HCD, *n* = 5); a hypercaloric diet group supplemented with *S. platensis* at doses of 25 (HCD+SP25, *n* = 5), 50 (HCD+SP50, *n* = 5), and 100 mg/kg (HCD+SP100, *n* = 5); and a hypercaloric diet group subsequently fed a standard diet (HCD+SD, *n* = 5). In the rats fed a hypercaloric diet, dietary supplementation with *S. platensis* effectively increased the number of erections while decreasing latency to initiate penile erection. Additionally, *S. platensis* increases NO bioavailability, reduces inflammation by reducing the release of contractile prostanoids, enhances the relaxation effect promoted by acetylcholine (ACh), restores contractile reactivity damage and cavernous relaxation, reduces reactive oxygen species (ROS), and increases cavernous total antioxidant capacity (TAC). Food supplementation with *S. platensis* thus restores erectile function in obese rats, reduces production of contractile prostanoids, reduces oxidative stress, and increases NO bioavailability. Food supplementation with *S. platensis* thus emerges as a promising new therapeutic alternative for the treatment of erectile dysfunction as induced by obesity.

## 1. Introduction

The close relationship between diet and health has been evidenced in many studies where the presence of bioactive molecules is described as influencing various metabolic pathways and systems within the organism. Abnormal eating habits represent a critical concern, promoting health disorders including cancer, diabetes, cardiovascular diseases, obesity, and sexual dysfunction [1, 2].

In recent decades, researchers have demonstrated a growing interest in natural sources of bioactive compounds such as fruits, vegetables, fish, herbs, and seaweeds as effective health promoters that can play an important and promising role in the prevention and treatment of disease [2–4]. Seaweed is an abundant source of bioactive metabolites, presenting many structures that are not found in terrestrial plants [5]. *Arthrospira platensis*, better known as *Spirulina platensis*, is known for its medicinal and nutritional potential which is often attributed to its complex chemical composition. Its biological and pharmacological activities are already described [6–8].

*Spirulina platensis* is a blue-green filamentous microalga, a multicellular photosynthetic cyanobacteria [9–11], often regarded as a high-quality natural superfood. It is rich in proteins, carbohydrates, fibers, polyunsaturated fatty acids (PUFAs), vitamins, and minerals [12–14] and constitutes an important source of bioactive compounds, such as chlorophyll, lutein, phycocyanin, *β*-carotene, fucoxanthins, phycobilins, and allophycocyanin [5, 15, 16]. These bioactive compounds are responsible for various anti-inflammatory, antioxidant [17–19], antihypertensive [20, 21], immunomodulative and anticancer [22, 23], antiobesity [24, 25], antidiabetic [26], antimicrobial [27], and reproprotective [28] properties. *S. platensis*, when used as a food supplement for humans and animals [29], is beneficial for management of diabetes, arthritis, allergies, obesity, cardiovascular disease, and even organic disorders such as erectile dysfunction (ED) [19, 30–33].

Erectile dysfunction is characterized as an inability to achieve and/or maintain adequate penile erection for satisfactory sexual intercourse [34]. ED is a symptomatic manifestation of various diseases, being the most prevalent sexual dysfunction affecting men after 40 years of age. It is estimated that more than 150 million men worldwide have some degree of erectile dysfunction, and this is projected to affect approximately 250 million men by 2025 [35, 36]. Several conditions are normally involved in the impairment of erectile function, such as high blood pressure, age, physical inactivity, dyslipidemia, diabetes, and obesity [37–39].

It has recently been shown that food supplementation with *S. platensis* promotes beneficial effects on the NO signaling pathway in the aorta of healthy rats [8], reduces oxidative stress and body adiposity in obese rats ileo [40], and prevents damage caused by a hypercaloric diet in erectile function [41, 42], highlighting the promising role of seaweed in the prevention of various organic and metabolic disorders.

Given the above, our study is aimed at investigating the modulatory effects of food supplementation with *Spirulina platensis* in reversing the damage caused by a hypercaloric diet (16 weeks) on the erectile function of rats and contributes to its development as a potential therapeutic agent for the treatment of obesity-induced erectile dysfunction.

## 2. Materials and Methods

### 2.1. Drugs

Calcium chloride dihydrate (CaCl_2_·2H_2_O), magnesium sulfate heptahydrate (MgSO_4_·7H_2_O), and glucose (C_6_H_12_O_6_) were purchased from Vetec (Rio de Janeiro, RJ, Brazil). Sodium bicarbonate (NaHCO_3_), sodium chloride (NaCl), and potassium chloride (KCl) were purchased from Nuclear (Porto Alegre, RS, Brazil). Monobasic potassium phosphate (KH_2_PO_4_), monobasic sodium phosphate (NaH_2_PO_4_), sodium hydroxide (NaOH), and hydrochloric acid (HCl) were purchased from Dinâmica (Diadema, SP, Brazil).

Phenylephrine (PHE) was purchased from Pfizer (USA). ACh, R-(-)-apomorphine, N*ω*-nitro-L-arginine methyl ester (L-NAME), indomethacin, tempol, apocinin, ethylenediamine tetraacetic acid (EDTA), MDA, and 1,1-diPHEnyl-2-picrylhydrazyl (DPPH) were purchased from Sigma-Aldrich (Brazil). To obtain the stock solutions, substances used in the functional experiments were dissolved and diluted in distilled water (indomethacin and apocinin were dissolved in absolute alcohol 96° GL), being, respectively, kept at 4 or -20°C. The carbogen mixture (95% O_2_ and 5% CO_2_) was obtained from White Martins (Brazil). All substances were weighed on an analytical balance, GEHAKA model AG 200 (Sao Paulo, SP, Brazil).

### 2.2. Animals

Wistar male rats (*Rattus norvegicus*), 2 months old and weighing approximately 150 g, were obtained from the Animal Production Unit (APU). The animals were maintained under controlled ventilation and temperature (21 ± 1°C) with water *ad libitum* in a 12 h light-dark cycle (lights on from 600 to 1800 h). The experimental procedures (being previously approved by the Ethics Committee on Animal Use of UFPB with certificate number 6061090318) were performed following guidelines for the ethical use of animals in applied etiology studies [43], and those of the Conselho Nacional de Controle de Experimentação Animal (in Brazil) [44].

### 2.3. Preparation and Supplementation with *Spirulina platensis*

*Spirulina platensis* in powder form was obtained from Bio-Engineering Dongtai Top Co., Ltd. (Nanjing, China) (Lot No. 20130320). To certify the extract, a sample was then analyzed by the Pharma Nostra Quality Control Laboratory (Anapolis, GO) (Lot No. 1308771A). Preparation of the powder was performed by Dilecta Manipulation Pharmacy (João Pessoa, PB) (Lot No. 20121025).

The *S. platensis* powder was dissolved in saline solution (NaCl 0.9%) at doses of 25, 50, and 100 mg/kg. The supplemented groups (at all doses) received administrations for 8 weeks [45]. Oral administration occurred daily between 1200 and 1400 h, using stainless steel needles for gavage (BD-12, Insight, Ribeirão Preto, SP) and 5 mL syringes accurate to 0.2 mL (BD, HIGILAB, Joao Pessoa, PB).

### 2.4. Groups and Diets

**Figure 1:**
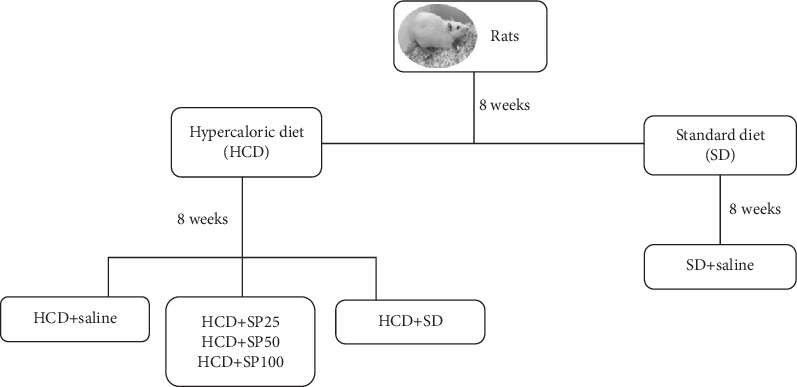
The rats were divided into 6 experimental groups and fed for a total period of 16 weeks. Initially, the animals were divided into two groups HCD = hypercaloric diet and SD = standard diet and fed for 8 weeks with their respective diets. Then, the HCD group was divided into three groups for another 8 weeks: HCD+saline (hypercaloric diet with saline solution); HCD+SP25, SP50, SP100 = hypercaloric diet supplemented with *Spirulina platensis* at doses of 25, 50, and 100 mg/kg; and an HCD+SD = hypercaloric diet group later fed a standard diet. The SD+saline group received saline solution for the final 8 weeks as well.

In phase I of the study (8 weeks), the animals were randomly divided into two groups (5 rats/group): rats given a standard diet (SD) of Presence® containing 7% moisture, 3% ashes, 23% protein, 63% carbohydrate, 4% lipids, with an energy density/g of 3.8 kcal; and rats fed a hypercaloric diet (HCD) consisting of the standard diet (Presence®), supplemented with milk chocolate, peanuts, and sweet biscuits at a ratio of 3 : 2 : 2 : 1 [32, 41]. The hypercaloric diet, containing 11% moisture, 5% ashes, 23% protein, 45% carbohydrate, and 16% lipid with an energy density of 4.2 kcal/g by weight, was prepared weekly and fed to the animals as granules [41]. In phase II, the SD and HCD groups fed their respective diets for another 8 weeks. The HCD group animals, however, were split into 3 experimental groups: rats fed a hypercaloric diet with saline solution HCD+saline; rats fed a hypercaloric diet and supplemented with *S. platensis* at doses of 25 (HCD+SP25), 50 (HCD+SP50), and 100 mg/kg (HCD+SP100); and rats fed a hypercaloric diet and later with the standard diet (HCD+SD); the SD group continued while receiving only saline as supplementation. All experimental groups were fed for a total of 16 weeks (Figure 1), and after this period, the animals were anesthetized with sodium thiopental (100 mg/kg body weight) mixed with lidocaine (10 mg/mL) and then euthanized being decapitated by guillotine.

### 2.5. Isolating the Corpus Cavernosum

The animals were euthanized by guillotine and the corpus cavernosum removed, being immersed in nutrient solution at room temperature and bubbled with a carbogen mixture. The penis was isolated near its attachment to the ischium bone, and the penile dorsal vein and urethra were removed. The corpus cavernosum was then separated into two 1 cm segments (approximately). After separation, the organ was suspended vertically through two stainless steel metal rods in an isolated organ bath (6 mL) containing Krebs-Ringer solution at 37°C. The upper stem was connected to the isometric force transducer, with the resting tension equivalent to 0.5 g [46]. The preparation was kept at rest for a period of 60 min for stabilization, with renewal of the nutrient solution every 15 min to avoid the influence of metabolites released by the organ to the environment [46, 47].

The Krebs-Ringer solution used presented a composition (in mM) of NaCl (118.4), KCl (4.7), CaCl_2_ (2.5), MgSO_4_ (1.2), KH_2_PO_4_ (1.17), NaHCO_3_ (25.0), and D-glucose (5.6). The pH was adjusted to 7.4 [48].

### 2.6. Evaluation of *In Vivo* Erectile Function

Each rat was placed in a glass box for 30 min (individually), receiving a subcutaneous dorsal region injection of R-(-)-apomorphine (80 *μ*g/kg) prepared in saline, and filmed for 30 min using two digital cameras.

From the images, the time of erection onset and the number of erections obtained by each animal were evaluated. Erections were considered events in which the animal's erect penis could be observed, accompanied by lordosis in which the animal is observed resting on its hind legs, tilting its body toward the genital area, while holding its penis with its front paws and licking for more than 5 s [49, 50]. The evaluation of erectile function *in vivo* was carried out after the end of 16 weeks of consumption of the diets for all experimental groups.

### 2.7. Contractile Reactivity Measurement

As previously described, the corpus cavernosum was assembled, and after the stabilization period (60 min), cumulative concentration-response curves for PHE (10^−8^ − 3 × 10^−3^ M) were obtained [46, 47]. Contractile reactivity was calculated using the maximum rat corpus cavernosum response amplitude found for the SD group. Comparisons were made between SD, HCD, HCD+SP25, HCD+SP50, HCD + SP100, and HCD+SD, using maximum effect values (*E*_max_) and the negative logarithm (base 10) of the PHE concentration producing 50% of the *E*_max_ (pEC_50_). This, being calculated from the cumulative concentration-response curves, was obtained.

### 2.8. Evaluation of NO and Cyclooxygenases

The corpus cavernosum was assembled as previously described. After stabilization, incubations (in distinct preparations) for a period of 30 min using a nonselective NOS inhibitor-L-NAME (10^−4^ M) [51] and a nonselective COX blocker-indomethacin (10^−5^ M) [52] were performed, and cumulative concentration-response curves for PHE (10^−8^ − 3 × 10^−3^ M) were obtained.

The contractile response of the corpus cavernosum in the presence of the inhibitors was calculated based on the mean amplitude of the curve obtained from the SD group. Contractile reactivity was assessed based on PHE *E*_max_ and pEC_50_ values obtained in the absence and presence of L-NAME and indomethacin in the separate preparations. The effects of the inhibitors on the cumulative concentration-response curve for PHE were compared between the SD, HCD, HCD+SP25, HCD+SP50, HCD+SP100, and HCD+SD treatments.

### 2.9. Relaxation Reactivity Measurement

The corpus cavernosum was assembled as previously described, and after the stabilization period, a contraction with PHE (10^−5^ M) was induced. Upon formation of the tonic component, ACh (10^−11^ − 3 × 10^−4^ M) was cumulatively added (in distinct preparations) to the organ bath [46, 47].

The relaxation response was expressed as the reverse percentage of the initial contraction produced by PHE. Comparisons were made between the SD, HCD, HCD+SP25, HCD+SP50, HCD+SP100, and HCD+SD groups based on the *E*_max_ and pEC_50_ values of the relaxation agents, being calculated from the cumulative concentration-response curves obtained.

### 2.10. Functional Assessment of Oxidative Stress

After stabilization, either apocinin (10^−4^ M), an NADPH oxidase inhibitor [53], or tempol (10^−3^ M), a superoxide dismutase (SOD) mimetic [54], was incubated in distinct preparations. A contraction was induced with PHE (10^−5^ M), and upon formation of the tonic component, ACh (10^−11^ − 3 × 10^−4^ M) was added to the bath.

The corpus cavernosum relaxation response to ACh, in the presence of apocinin or tempol, was thus calculated based on the maximum contraction amplitude; relaxation reactivity was evaluated according to the ACh *E*_max_ and pEC_50_ values in the absence and presence of the inhibitors and compared between the SD, HCD, and HCD+SP50 groups.

### 2.11. Lipid Peroxidation Assessment

Following euthanasia, the rats' corpus cavernosum was isolated and frozen at -20°C until homogenate preparation. In this procedure, the organ was weighed, macerated, and homogenized with a 10% KCl solution in a 1 : 1 ratio. The samples were then centrifuged at 1198 g for 10 min, and the supernatant obtained was separated for analysis.

Lipid peroxidation was measured using the chromogenic product of 2-thiobarbituric acid (TBA) reaction with malondialdehyde (MDA); a product formed as a result of membrane lipid peroxidation [55]. Tissue homogenate (250 *μ*L) was incubated in a water bath at 37°C for 60 min, and the samples were then precipitated with 400 *μ*L of 35% perchloric acid and centrifuged at 26,295 g for 10 min at 4°C. The supernatant was transferred to new Eppendorf tubes, and 400 *μ*L of 0.6% thiobarbituric acid was added. This was followed by incubation at 95-100°C for 30 min. After cooling, the samples were read at 532 nm. The concentrations of malondialdehyde in the tissue samples (30, 20, 15, 12, 10, 8.57, 6.6, 5.45, and 4.61 mmol/L tissue) were determined using an MDA standard curve constructed using a solution standard (1 *μ*L of 1,1,3,3-tetramethoxypropane in 70 mL of distilled water). The tissue absorbance values obtained were normalized to the dry weight present in each given sample volume.

### 2.12. Antioxidant Activity Assay

The procedure was based on the method described by Brand-Williams et al. [56], where 1.25 mg of DPPH (1,1-diPHEnyl-2-picrylhydrazyl radical) was dissolved in 100 mL of ethanol, and kept under refrigeration and protected from light (aluminum paper or amber glass). Then, 3.9 mL of this DPPH solution was added together with 100 *μ*L of the supernatant homogenate to appropriate centrifuge tubes, vortexed, and allowed to stand for 30 min. They were centrifuged at 1207 g for 15 min at 20°C, and the absorbance of the supernatant was read at 515 nm. The results were expressed as a percentage of oxidation inhibition where AOA (antioxidant activity) = 100 − ((DPPH · R) T/(DPPH · R) B 100), where (DPPH · R) and (DPPH · R) B correspond to the concentration of DPPH · remaining after 30 min, measured in the sample (T) and blank (B) prepared with distilled water. The tissue samples were homogenized with 10% KCl at a 1 : 1 ratio. The absorbance values obtained for the tissue were normalized to the dry weight present in a given sample volume.

### 2.13. Statistical Analysis

The functional results obtained were expressed as mean and standard error of the mean (S.E.M.) (*n* = 5) and statistically analyzed for intergroup comparison using Student's *t*-test. The results were statistically analyzed using two-way analysis of variance (ANOVA) followed by Bonferroni's posttest. The differences between the means were considered significant when *p* < 0.05. The pCE_50_ values were calculated using nonlinear regression [57], and *E*_max_ was obtained by averaging the maximum percentages of contraction or relaxation. All results were analyzed using GraphPad Prism version 5.01 (GraphPad Software Inc., San Diego CA, USA).

## 3. Results

### 3.1. *In Vivo* Effects of Hypercaloric Diet Intake and Food Supplementation with *S. platensis* on Erectile Function

**Figure 2:**
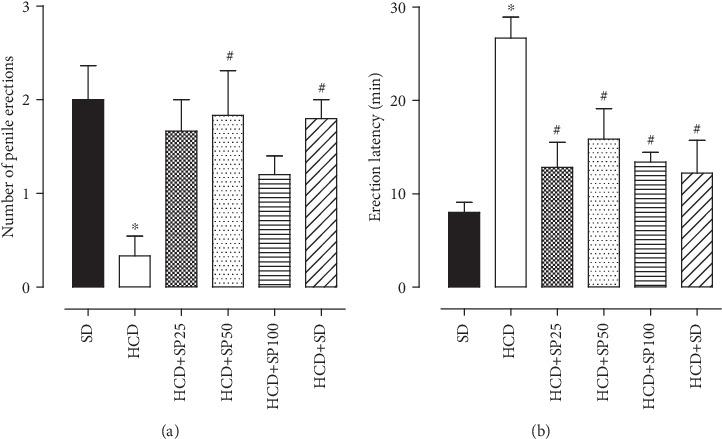
Number of penile erections (a) and latency to penile erection (b) in rats for the SD, HCD+SP25, HCD+SP50, HCD+SP100, and HCD+SD groups. Columns and vertical bars, respectively, represent the mean and S.E.M. (*n* = 5). ANOVA one-way followed by Tukey's posttest. ^∗^*p* < 0.05 (SD *vs.* HCD) and ^#^*p* < 0.05 (HCD *vs.* HCD+SP25, HCD+SP50, HCD+SP100, and HCD+SD). SD = group fed the standard diet; HCD = group fed a hypercaloric diet; HCD+SP25, HCD+SP50, and HCD+SP100 = groups fed a hypercaloric diet and, respectively, supplemented with *S. platensis* at the doses of 25, 50, and 100 mg/kg; HCD+SD = hypercaloric diet group later fed a standard diet.

In the HCD group (0.3 ± 0.2), it was observed that the number of penile erections was lower than that in the SD group (2.0 ± 0.4). When rats consumed the hypercaloric diet and were supplemented with *S. platensis* at doses of 25 (1.7 ± 0.3) and 100 mg/kg (1.2 ± 0.2), no difference was observed as compared to the HCD group. However, the HCD+SP50 (1.8 ± 0.5) and HCD+SD (1.8 ± 0.2) groups presented an increase in the number of penile erections as compared to the hypercaloric diet group (0.3 ± 0.2) (Figure 2(a)).

The latency to obtain penile erection in the HCD group (26.7 ± 2.2 min) was higher than that in the SD group (8.0 ± 1.0 min). In the HCD+SP25 (12.8 ± 2.6 min), HCD+SP50 (15.3 ± 3.3 min), and HCD+SP100 (13.4 ± 1.0 min) groups (the three algal doses tested), as well as in the HCD+SD group (12.2 ± 3.5 min), a reduced latency time was observed as compared to the HCD group (Figure 2(b)).

### 3.2. Effects of a Hypercaloric Diet with *S. platensis* Supplementation on the Contractile Reactivity of Corpus Cavernosum to PHE

**Figure 3:**
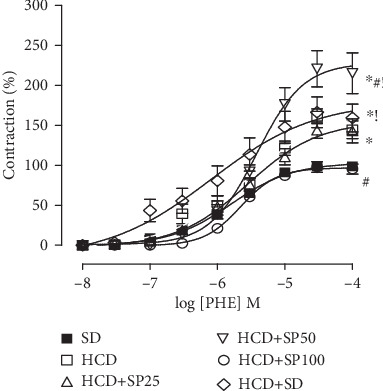
Cumulative concentration-response curves for PHE, in SD (■), HCD (□), HCD+SP25 (∆), HCD+SP50 (∇), HCD+SP100 (○), and HCD+SD (◊) groups, in isolated corpus cavernosum. The symbols and vertical bars, respectively, represent the mean and S.E.M. (*n* = 5). ANOVA one-way followed by Tukey's posttest. ^∗^*p* < 0.05 (SD *vs.* HCD, HCD+SP50, or HCD+SD), ^#^*p* < 0.05 (HCD *vs.* HCD+SP25, HCD+SP50, HCD+SP100, or HCD+SD), ^!^*p* < 0.05 (HCD+SP25 *vs.* HCD+SP50 or HCD+SD), and ^&^*p* < 0.05 (HCD+SP100 *vs.* HCD+SD). PHE = phenylephrine; SD = standard diet group; HCD = hypercaloric diet group; HCD+SP25, HCD+SP50, and HCD+SP100 = hypercaloric diet groups, respectively, supplemented with *S. platensis* at 25, 50, and 100 mg/kg; HCD+SD = hypercaloric diet group later fed a standard diet.

An increase in the maximum effect of PHE was observed in the group that consumed a hypercaloric diet (*E*_max_ = 161.5 ± 11.2%; pEC_50_ = 5.6 ± 0.1), when compared to the SD group (*E*_max_ = 100%; pEC_50_ = 5.8 ± 0.04) (Figure 3).

In rats fed a hypercaloric diet supplemented with *S. platensis* at a dose of 25 mg/kg (*E*_max_ = 147.3 ± 13.4%; pEC_50_ = 5.4 ± 0.1), the contractile reactivity of the corpus cavernosum was unaltered by PHE. However, supplementation at a dose of 50 mg/kg (*E*_max_ = 224.2 ± 22.2%; pEC_50_ = 5.6 ± 0.1) increased the contractile efficacy of PHE when compared to the SD, HCD, and HCD+SP25 groups. However, in the *S. platensis* supplemented group (100 mg/kg), a reduction in the contractile efficacy of PHE (*E*_max_ = 98.0 ± 6.8%; pEC_50_ = 5.6 ± 0.04) was observed as compared to that in the HCD group (*E*_max_ = 161.5 ± 9.3%; pEC_50_ = 5.8 ± 0.2) (Figure 3). In the hypercaloric diet group that subsequently consumed a standard diet (*E*_max_ = 166.4 ± 19.3%), the contractile efficacy of PHE was higher as compared to that in the SD group (*E*_max_ = 100%) or the HCD+SP25 group (*E*_max_ = 147.3 ± 13.4%) (Figure 3).

### 3.3. Effects of a Hypercaloric Diet with *S. platensis* Supplementation on NO and Cyclooxygenase (COX) Pathways

In the SD group, an increase in the maximum effect and contractile potency of PHE (*E*_max_ = 153.8 ± 17.9%; pEC_50_ = 5.5 ± 0.04) in the presence of L-NAME (a nonselective inhibitor of NOS) was observed as compared to the SD group in the absence of L-NAME (*E*_max_ = 100%; pEC_50_ = 5.8 ± 0.04). In the presence of indomethacin (a nonselective COX blocker) in the SD group, no change was observed in the PHE curve (*E*_max_ = 90.5 ± 2.6%; pEC50 = 5.6 ± 0.05) as compared to its absence (*E*_max_ = 100%; pEC_50_ = 5.8 ± 0.04) (Table 1).

When analyzing the PHE curve in the presence of L-NAME, in the HCD group, a twofold reduction in contractile efficacy was observed (*E*_max_ = 80.0 ± 9.5%; pEC_50_ = 5.6 ± 0.2) as compared to the absence of L-NAME (*E*_max_ = 161.5 ± 9.3%; pEC_50_ = 5.4 ± 0.08). A reduction in contractile efficacy for indomethacin in the absence of L-NAME (*E*_max_ = 90.5 ± 2.6%; pEC_50_ = 5.6 ± 0.05) was also observed (Table 1).

In rats fed a hypercaloric diet supplemented with *S. platensis* at 50 mg/kg dose, both the efficacy and contractile potency of PHE were unaltered in the presence of both L-NAME (*E*_max_ = 179.8 ± 10.4%; pEC_50_ = 5.6 ± 0.08) and indomethacin (*E*_max_ = 183.3 ± 12.0%; pEC_50_ = 5.5 ± 0.08) as compared to their absences (Table 1).

In the HCD+SP100 group, the contractile efficacy of PHE (*E*_max_ = 186.7 ± 18.6%; pEC_50_ = 5.4 ± 0.1) doubled in the presence of L-NAME. Similarly, the contractile efficacy of the agonist was also augmented in the presence of indomethacin (*E*_max_ = 216.7 ± 10.3%; pEC_50_ = 5.4 ± 0.1) as compared to its absence (*E*_max_ = 98.0 ± 6, 8%; pEC_50_ = 5.6 ± 0.04) (Table 1).

In the HCD+SD group rats, a twofold reduction in PHE contractile efficacy was observed in the presence of L-NAME without a change in potency (*E*_max_ = 80.5 ± 14.4%; pEC50 = 5.9 ± 0.1). In the presence of indomethacin, contractile efficacy diminished by 3.8 fold (*E*_max_ = 43.8 ± 3.1%; pEC_50_ = 5.7 ± 0.1) as compared to the absence of these inhibitors (*E*_max_ = 166.4 ± 19.3%; pEC_50_ = 6.0 ± 0.1) (Table 1).

### 3.4. Effect of Hypercaloric Diet and *S. platensis* Supplementation on Corpus Cavernosum Relaxation Reactivity to ACh

**Figure 4:**
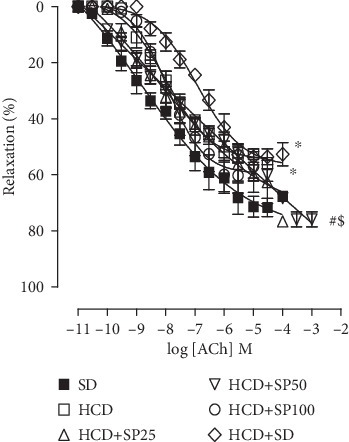
Cumulative ACh concentration-response curves in isolated corpus cavernosum: SD (■), HCD (□), HCD+SP25 (∆), HCD+SP50 (∇), HCD+SP100 (○), and HCD+SD groups (◊). The symbols and vertical bars represent the mean and S.E.M., respectively (*n* = 5). ANOVA one-way followed by Tukey's posttest. ^∗^*p* < 0.05 (SD *vs.* HCD), ^#^*p* < 0.05 (HCD *vs.* HCD+SP50), and ^$^*p* < 0.05 (HCD+SD *vs.* HCD+SP50). ACh = acetylcholine. SD = standard diet group; HCD = hypercaloric diet group; HCD+SP25, HCD+SP50, and HCD+SP100 = hypercaloric diet groups supplemented with *S. platensis*, respectively, at 25, 50, and 100 mg/kg; HCD+SD = hypercaloric diet group later fed a standard diet.

The relaxation efficacy of ACh was lower in the HCD group (*E*_max_ = 53.5 ± 1.5%; pEC_50_ = 7.9 ± 0.1) as compared to that in the SD group (*E*_max_ = 72.7 ± 3.3%; pEC50 = 8.2 ± 0.2). In the hypercaloric diet groups, supplementation with *S. platensis* at doses of 25 (*E*_max_ = 57.2 ± 5.7%; pEC50 = 8.1 ± 0.3) and 100 mg/kg (*E*_max_ = 60.2 ± 6.2%; pEC50 = 7.8 ± 0.2) did not alter ACh-promoted relaxation as compared to that in the HCD group (*E*_max_ = 53.5 ± 1.5%; pEC50 = 8.2 ± 0.2) (Figure 4).

Supplementation with *S. platensis* algae at 50 mg/kg (pEC_50_ = 7.1 ± 0.2) in the HCD+SD group (pEC_50_ = 7.0 ± 0.1) reduced ACh contractile potency as compared to that in the HCD group (pEC_50_ = 7.9 ± 0.1). The best relaxation efficacy for this agonist was observed in the HCD+SP50 group (*E*_max_ = 75.9 ± 2.7%), this as compared to that in the HCD group (*E*_max_ = 53.5 ± 1.5%). However, ACh relaxation was reduced in the HCD+SD group (*E*_max_ = 53.6 ± 2.8%) when compared to that in the SD group (*E*_max_ = 72.7 ± 3.3%) (Figure 4).

### 3.5. Effect of Hypercaloric Diet with *S. platensis* Food Supplementation on Functional Oxidative Stress

**Figure 5:**
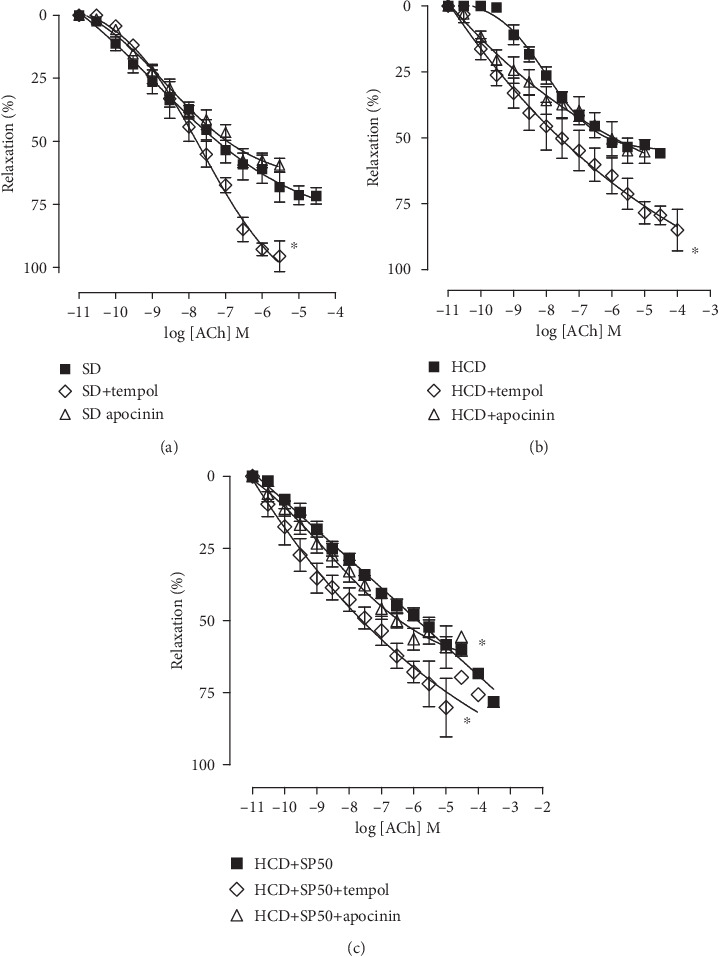
Cumulative concentration-response curves for ACh in isolated rat corpus cavernosum in the absence (■) and presence of tempol (◊) and apocinin (*Δ*) for the SD (a), HCD (b), and HCD+SP50 (c) groups. The symbols and vertical bars represent the mean and S.E.M., respectively (*n* = 5). ANOVA one-way followed by Tukey's posttest. ^∗^*p* < 0.05 (absence *vs.* tempol and apocinin). ACh = acetylcholine. SD = standard diet group; HCD = hypercaloric diet group; HCD+SP50 = hypercaloric diet group supplemented with *S. platensis* 50 mg/kg.

In the presence of tempol, a SOD mimetic, the SD group presented greater ACh relaxation efficacy without a change in potency (*E*_max_ = 90.7 ± 6.9%; pEC_50_ = 8.1 ± 0.3) as compared to its absence (*E*_max_ = 72.7 ± 3.3%; pEC_50_ = 8.2 ± 0.2). In the presence of apocinin, a NADPH oxidase complex inhibitor, no change in efficacy or relaxation potency (*E*_max_ = 57.6 ± 2.8%; pEC_50_ = 8.5 ± 0.2) was observed when compared to its absence (*E*_max_ = 72.7 ± 3.3%; pEC_50_ = 8.2 ± 0.2) (Figure 5(a)).

In the presence of tempol, the relaxation effect in the HCD group was increased (*E*_max_ = 84.4 ± 6.6%; pEC_50_ = 8.1 ± 0.4) without change in potency when compared to its absence (*E*_max_ = 53.5 ± 1.5%; pEC_50_ = 7.9 ± 0.1). Additionally, there was no change in efficacy or relaxation potency in the presence of apocinin (*E*_max_ = 55.2 ± 4.5%; pEC_50_ = 8.5 ± 0.3) as compared to the inhibitor absence curve (*E*_max_ = 53.5 ± 1.5%; pEC_50_ = 7.9 ± 0.1) (Figure 5(b)).

Supplementation with *S. platensis* at 50 mg/kg did not alter the relaxation efficacy of ACh either in the presence of tempol (*E*_max_ = 78.4 ± 5.8%) or apocinin (*E*_max_ = 62.3 ± 1.8%) as compared to their absence (*E*_max_ = 53.5 ± 1.5%). However, the relaxation response was potentiated in the presence of both tempol and apocinin (pEC_50_ = 8.4 ± 0.3 and 8.2 ± 0.2, respectively) as to its absence (pEC50 = 7.1 ± 0.2) (Figure 5(c)).

### 3.6. Effect of Hypercaloric Diet with *S. platensis* Food Supplementation on Lipid Peroxidation

**Figure 6:**
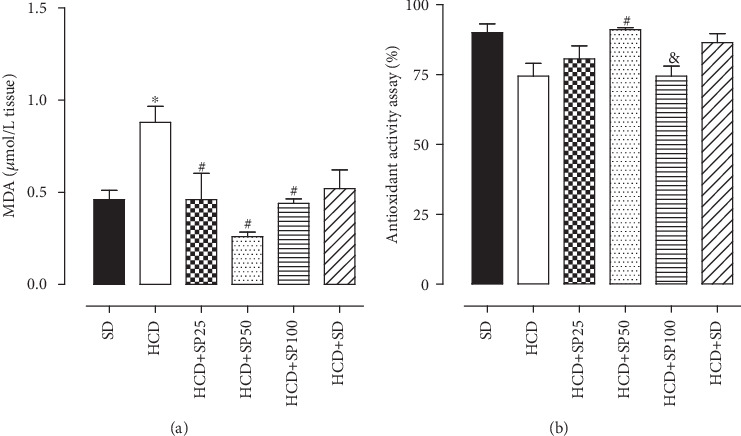
MDA concentrations from an antioxidant activity assay in isolated rat corpus cavernosum for the SD, HCD, HCD+SP25, HCD+SP50, HCD+SP100, and HCD+SD groups. Columns and vertical bars represent the mean and S.E.M., respectively (*n* = 5). ANOVA one-way followed by Tukey's posttest. ^∗^*p* < 0.05 (SD *vs.* HCD) and ^#^*p* < 0.05 (HCD *vs.* HCD+SP25, HCD+SP50, and HCD+SP100). SD = standard diet group; HCD = hypercaloric diet group; HCD+SP25, HCD+SP50, and HCD+SP100 = hypercaloric diet groups, respectively, supplemented with *S. platensis* 25, 50, and 100 mg/kg; HCD+SD = hypercaloric diet group later fed a standard diet.

The HCD group (0.9 ± 0.40 *μ*mol/L) presented a higher MDA concentration as compared to the SD group (0.5 ± 0.05 *μ*mol/L). However, supplementation with *S. platensis* at doses of 25 (0.5 ± 0.1 *μ*mol/L), 50 (0.3 ± 0.02 *μ*mol/L), and 100 mg/kg (0.4 ± 0 02 *μ*mol/L) reduced MDA levels in relation to both the HCD and SD groups. There was no difference in MDA concentration in the corpus cavernosum of the HCD+SD group (0.5 ± 0.1 *μ*mol/L) as compared to either the HCD group or the SD group (Figure 6(a)).

No difference in total antioxidant activity was observed between the isolated corpus cavernosum of rats fed the standard diet (90.0 ± 3.1%) and those fed a hypercaloric diet (74.4 ± 4.6%). In rats fed a hypercaloric diet supplemented with *S. platensis* at a dose of 50 mg/kg (91.0 ± 0.8%), there was an increase in the organ's total antioxidant activity as compared to the HCD group. A difference was also observed between HCD+SP50 and HCD+SP100 (respectively, 91.0 ± 0.8 and 74.4 ± 3.5%) (Figure 6(b)).

## 4. Discussion

In the present study, we investigated evidence of erectile dysfunction development in Wistar rats as induced by consumption of a hypercaloric diet. It was shown that hypercaloric food intake resulted in reduced erectile function, increased contractile efficacy, and reduced corpus cavernosum relaxation, with increased release of contractile prostanoids and ROS synthesis. In contrast, the deleterious effects were restored by food supplementation with *Spirulina platensis* through mechanisms involving NO, COX, and oxidative stress pathways.

Male Wistar rats were fed a hypercaloric diet for 16 weeks, the first 8 weeks being fed only with the standard diet or the hypercaloric diet (417.0 kcal/100 g), the following 8 weeks included alimentary supplementation with *S. platensis* at 25, 50, and 100 mg/kg. Additionally, in order to simulate a dietary reeducation process, another group of animals was fed a hypercaloric diet during the first 8 weeks being replaced by the standard diet during the following 8 weeks.

In order to evaluate the rats' erectile function, R-(-)-apomorphine, an inducer of penile erection, was used. The substance presents high affinity for D_2_-like receptors present in nucleoparaventricular oxytocinergic neurons of the hypothalamus [50, 58].

Erectile function in the groups fed a hypercaloric diet presented reductions in the number of penile erections and an increase in latency to start the erection as compared to the SD group, confirming development of ED [59]. After *S. platensis* supplementation, the animals demonstrated an increase in the number of penile erections (at the dose of 50 mg/kg), and a reduction in onset times to initiate penile erection (at all doses analyzed; 25, 50, and 100 mg/kg). Moreover, the HCD+SD group presented an increase in the number of penile erections as well as a reduction in latency to initiate penile erection, demonstrating the expected improvement in erectile function. Although less effective than supplementation with *S. platensis*, such “lifestyle” changes, specifically towards healthy eating habits and physical activity contributing to the improvement of sexual function (Figure 1).

The relationship between penile erection and flaccidity is directly associated with the existing combination of contraction and relaxation processes in cavernous smooth muscle cells [60]. To evaluate contractile and relaxation response in the corpus cavernosum, cumulative concentration-response curves for PHE and ACh were performed for Wistar rats. When comparing the PHE curve between the HCD and SD groups, increased contractile efficacy was observed in the HCD group, thus, demonstrating the deleterious influence of a hypercaloric diet on the mechanisms that favor cavernous contractility (Figure 3) [61, 62].

The relaxation efficacy of ACh in the HCD group was decreased when compared to the SD group (Figure 5). Previous studies have demonstrated a correlation between endothelial dysfunction and reduced endothelium-dependent relaxation in the corpus cavernosum of obese mice [63], diabetic rats [64], and elderly rats [62].

Interestingly, when supplemented with 50 mg/kg of *S. platensis*, increased PHE contractile efficacy was observed. Rats fed with the same hypercaloric diet for 16 weeks, with *S. platensis* supplementation at the same dose, revealed increased calcium pathway sensitization through positive modulation of the Rho/ROCK pathway in intestinal smooth muscle, contributing to the maintenance of muscle contraction [65]. However, supplementation with a dose of 100 mg/kg led to a decrease in PHE contractile efficacy, demonstrating a complete reversal of the deleterious effects of the hypercaloric diet, as well as normalizing the erectile function of the animals (Figure 3). It can be inferred that the alga may reduce cavernous contraction pathway steps, while activating signaling pathway steps that result in muscle relaxation.

In contrast, when compared to the HCD group, no difference in contractile and relaxation efficacy was observed in the group fed a hypercaloric diet and later a standard diet. This suggests that changes in dietary habits, specifically the diet alone, are unable to reverse the damage caused by obesity on cavernous responsiveness (Figure 5).

Dietary supplementation with *S. platensis* at a dose of 50 mg/kg increased the relaxation efficacy of ACh, exceeding the relaxation observed in rats fed a standard diet alone (Figure 5). Given these results, it can be inferred that *S. platensis* supplements may positively modulate both NO signaling pathways and prostanoids, since the alterations and deleterious effects triggered by the consumption of a hypercaloric diet on relaxation reactivity were restored.

The pathogenesis of erectile dysfunction is related to endothelial dysfunction. The dysfunction is associated with decreased nitric oxide synthase (NOS), and consequent reduction of NO availability in the corpus cavernosum, as well as with an imbalance between the production of contractile and relaxation prostanoids [66–68].

Based on this information, L-NAME, a nonselective NOS inhibitor [69], and indomethacin, a nonselective COX blocker, were both incubated in distinct preparations [70]. In the SD group, an increase in the efficacy and contractile potency of PHE was demonstrated in the presence of L-NAME. In the HCD group, a twofold reduction in contractile efficacy was revealed (Figure 4), suggesting that NO formation hinders contraction of the corpus cavernosum, confirming its potent vasodilator role. In addition, the reduced availability of NO in the rats fed a hypercaloric diet increases contraction through endothelial dysfunction and also suggests that NO reacts with superoxide anions originating in increased body fat, to form peroxynitrite, which is a potent contractile factor.

Evaluating the rats consuming hypercaloric diet, a decrease in the contraction curve induced by PHE in the presence of the COX inhibitor when compared to its absence was observed (Figure 4), inferring that in this system, consumption of a hypercaloric diet modifies synthesis and/or release of lipid mediators through increased contractile prostanoid production (over that of relaxants), which is evidenced in the blocker contraction curve reduction evidenced in the presence of indomethacin [71].

Dietary supplementation with *S. platensis* at a dose of 100 mg/kg promoted an increase in contraction in the presence of L-NAME, which may infer that *S. platensis* acts as a potent antioxidant, positively modulating NO pathways, increasing the substrate production and expression of NOS, as well as NO itself, consequently, removing the superoxide anion free radical that favors vasoconstriction. Thus, NO bioavailability would increase, augmenting its vasodilator effect making contractions more difficult [8, 41, 72].

When the animals' feed was supplemented with *S. platensis* at 50 and 100 mg/kg, an increase in the PHE curve was observed in the presence of indomethacin (Figure 4). It is suggested that *S. platensis* might well be promoting an increase in the synthesis of relaxation prostanoids which hinder contraction. Thus, the alga, besides positively modulating the NO pathway, restores deleterious hypercaloric dietary effects on the prostanoid pathway, confirming the beneficial effect of *S. platensis* on erectile function.

Excessive free radical production is triggered by increased body adiposity and contributes to vascular damage, causing reduced erectile function [73, 74]. Given this, and the importance of oxidative stress in the development of ED (especially through the influence of free radicals on contractile and cavernous relaxation reactivity), it was hypothesized that consumption of a hypercaloric diet together with *S. platensis* supplementation in a dose that enhanced the effect of ACh (50 mg/kg) would alter relaxation of the corpus cavernosum through ROS modulation.

The preparations were incubated with apocinin, a nonselective NADPH oxidase inhibitor, and tempol, a superoxide dismutase (SOD) mimetic, previously cited as inducing relaxation with ACh [75–77]. In the SD group, no change in efficacy or potency in the ACh relaxation curve was observed in the presence of apocinin (Figure 5), inferring that ACh relaxation does not involve formation of the superoxide anion through the NADPH oxidase system. In the same group, in the presence of tempol (a SOD mimetic responsible for reducing superoxide anion levels), it was found that the relaxation promoted by ACh had increased (Figure 5). Thus, in a normal physiological system, the production and presence of superoxide anions are directly related to reductions in relaxation efficacy, hindering the relaxation of the rat corpus cavernosum [62, 78].

Similarly, when analyzing the hypercaloric diet rats, the ACh relaxation curve in the presence of apocinin and tempol was unchanged when compared to their absence. Thus, it is suggested that dietary change does not promote changes in the production of superoxide anion by NADPH oxidase, since in the absence of apocinin and tempol, both SD and HCD group relaxation curves overlap with their ACh relaxation curves. However, the possibility of ROS production involving alternative pathways in this complex cannot be ruled out.

Food supplementation with *S. platensis* at the 25, 50, and 100 mg/kg doses reduced oxidative stress markers and MDA levels in the corpus cavernosum, restoring the damage caused by the hypercaloric diet on the ROS pathway (Figure 6). Continuous production of free radicals during metabolic processes results in development of antioxidant defense mechanisms which limit intracellular levels of the reactive species to control cell damage and death [79–81]. Measurement of total antioxidant capacity helps to assess nutritional, physiological, and environmental redox balance factors in both humans and animals [82–84].

It was also demonstrated that the total antioxidant capacity of the corpus cavernosum was reduced in the HCD group, suggesting that increased body adiposity directly contributes to increased free radical production, and increased ROS synthesis, and that antioxidant systems are enhanced when there is an increase in the production of these reactive species [85, 86]. Food supplementation with *S. platensis* at a dose of 50 mg/kg promoted an increase in total antioxidant capacity, which justifies reduction of tissue MDA levels in these animals, while confirming the potent antioxidant effects promoted by the algae (Figure 6(b)).

## 5. Conclusion

**Figure 7:**
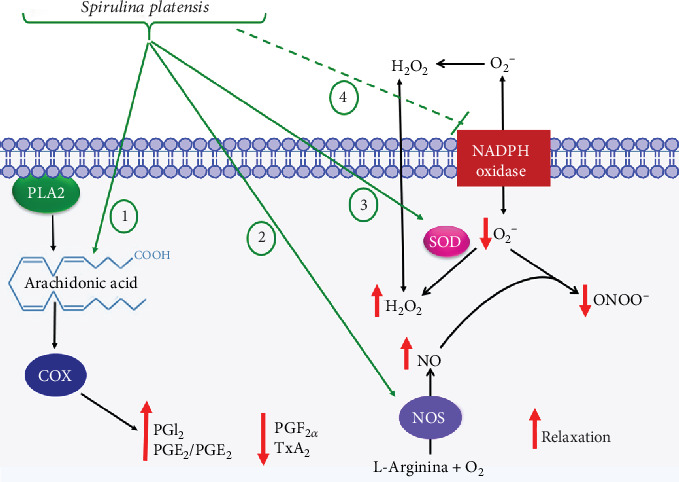
Modulation of cavernous muscle reactivity in Wistar rats through *S. platensis* supplementation of feed: 1: activation of the COX pathway, with increased production of relaxation prostanoids and consequent reduction of contractile prostanoids; 2: NOS activation with increased NO bioavailability; 3: activation of the enzyme SOD with consequent decrease of O_2_^−^, formation of ONOO^−^, and increase in H_2_O_2_ concentration; and 4: inhibition of the NADPH oxidase complex, with consequent decrease in O_2_^−^ formation.

This study evaluated the effects and therapeutic potential of dietary supplementation with *Spirulina platensis* (a kelp described in the literature as an important source of bioactive compounds), on *in vivo* erectile function, contractile and relaxation reactivity, and oxidative stress in cavernous smooth muscle. In conclusion, chronic dietary supplementation (*in vivo*) with *S. platensis* promoted a greater number of erections and reduced latency times to erection as compared to the HCD group, altered cavernous smooth muscle reactivity, and resulted in decreased contractile responsiveness to PHE. ACh further increases this relaxation response and reduces oxidative stress in obese rats. The mechanisms underlying these effects include increased production of relaxation prostanoids, contractile reduction, increased NO bioavailability, reduced lipid peroxidation, reduced levels of ROS, and increased antioxidant activity in the corpus cavernosum (Figure 7).

## Figures and Tables

**Table 1:** *E*_max_ and pCE50 values for PHE in the absence and presence of L-NAME and indomethacin, from the isolated corpus cavernosum of rats in the SD, HCD, HCD+SP50, HCD+SP100, and HCD+SD groups.

Groups/PHE	Absence	L-NAME	Indomethacin
SD			
*E*_max_ (%)	100	153.8 ± 17.9^∗^	90.5 ± 2.6^#^
pCE_50_	5.8 ± 0.04	5.5 ± 0.04^∗^	5.6 ± 0.05^∗^
HCD			
*E*_max_ (%)	161.55 ± 9.3	80.0 ± 9.5^∗^	6.0 ± 0.05^∗^
pCE_50_	5.6 ± 0.2	5.4 ± 0.08	5.7 ± 0.1
HCD+SP50			
*E*_max_ (%)	224.2 ± 22.2	179.8 ± 10.4	183.3 ± 12.0
pCE_50_	5.4 ± 0.07	5.6 ± 0.08	5.5 ± 0.08
HCD+SP100			
*E*_max_ (%)	98.0 ± 6.8	186.7 ± 18.6^∗^	216.7 ± 10.3^∗^
pCE_50_	5.6 ± 0.04	5.4 ± 0.1	5.4 ± 0.1
HCD+SD			
*E*_max_ (%)	166.4 ± 19.3	80.5 ± 14.4^∗^	43.8 ± 3.1^∗^
pCE_50_	6.0 ± 0.1	5.9 ± 0.1	5.7 ± 0.1

The symbols and vertical bars represent the mean and S.E.M., respectively (*n* = 5). ANOVA one-way followed by Tukey's posttest. ^∗^*p* < 0.05 (absence *vs.* L-NAME and indomethacin) and ^#^*p* < 0.05 (L-NAME *vs.* indomethacin). L-NAME = N*ω* nitro L-arginine methyl ester. PHE = phenylephrine; SD = standard diet group; HCD = hypercaloric diet group; HCD+SP50 and HCD+SP100 = hypercaloric diet groups supplemented with *S. platensis* 50 and 100 mg/kg, respectively; HCD+SD = hypercaloric diet group later fed a standard diet.

## Data Availability

The hypothesis and review data used to support the findings of this study are included within the article.

## Abbreviations

ACh: Acetylcholine
COX: Cyclooxygenase
EDTA: Ethylenediamine tetraacetic acid
ED: Erectile dysfunction
*E*_max_: Maximum effect
HC: Hypercaloric diet group
HCD+SD: Hypercaloric diet group and later fed standard diet
HCD+SP25: Hypercaloric diet group supplemented with SP25
HCD+SP50: Hypercaloric diet group supplemented with SP50
HCD+SP100: Hypercaloric diet group supplemented with SP100
L-NAME: N(*ω*)-Nitro-L-arginine methyl ester
MDA: Malondialdehyde
NO: Nitric oxide
NOS: Nitric oxide synthase
ONOO: Peroxynitrite
H_2_O_2_: Hydrogen peroxide
PGE_1_/PGE_2_: Prostaglandin E1/E2
PHE: Phenylephrine
PLA_2_: Phospholipase A2
PGF2*α*: Prostaglandin F2*α*
PGI_2_: Prostacyclin
ROS: Reactive oxygen species
S.E.M.: Standard error of the mean
SD: Standard diet group
SOD: Superoxide dismutase
TBARS: Thiobarbituric acid reactive substances
TxA_2_: Thromboxane A2.
